# Supplementary figures and images for: From the Clinic to the Bench and Back Again in One Dog Year: How a Cross-Species Pipeline to Identify New Treatments for Sarcoma Illuminates the Path Forward in Precision Medicine
Source: Front Oncol. 2020 Feb 11;10:117. doi: 10.3389/fonc.2020.00117 (PMC7026496; doi:10.3389/fonc.2020.00117)

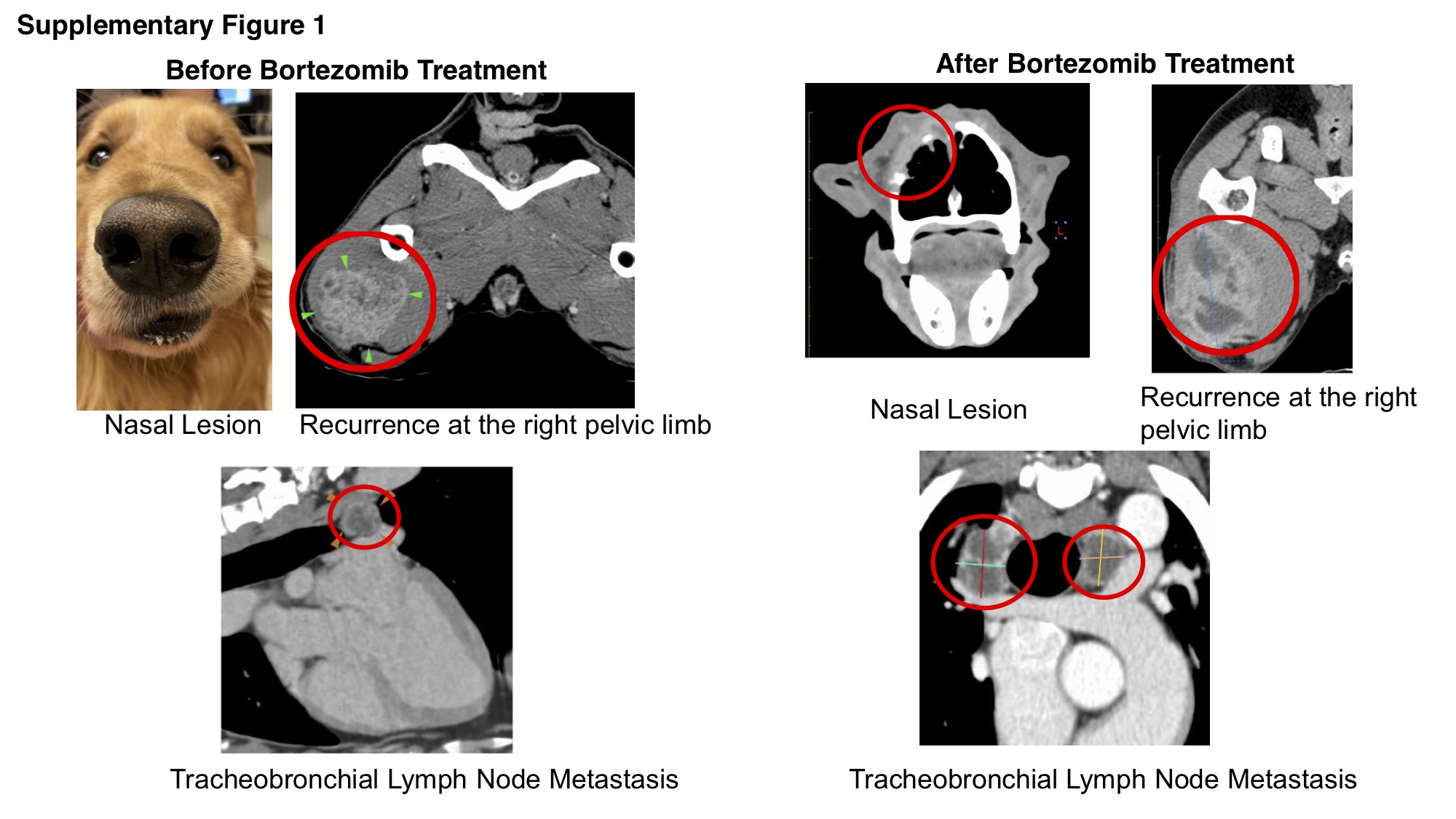

Supplement: Supplementary file 2 [file Image_1.JPEG]

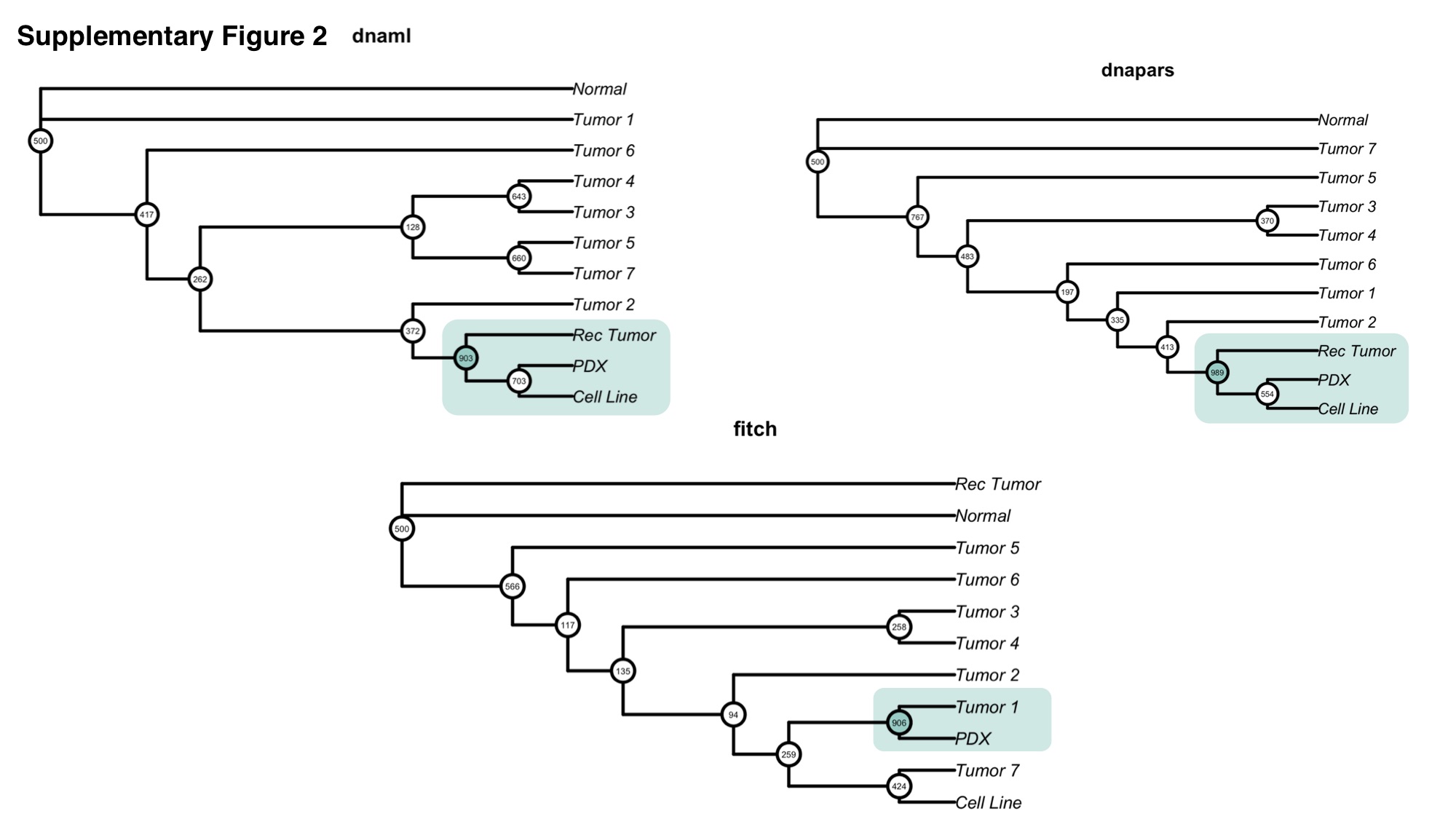

Supplement: Supplementary file 3 [file Image_2.JPEG]
